# Life with pets study: lower income veterinary clients' perception of pets' quality of life

**DOI:** 10.3389/fvets.2024.1422359

**Published:** 2024-07-17

**Authors:** Elizabeth B. Strand, Kristel Scoresby, Hannah Walker, Ana Hernandez, Veronica Accornero, Lori Messinger, Pamela Linden, Chesney Ward, Matthew P. Knight, Haley Engelman, Kristin Moore, Margaret Ruth Slater

**Affiliations:** ^1^Colleges of Social Work and Veterinary Medicine, Center for Veterinary Social Work, University of Tennessee, Knoxville, TN, United States; ^2^College of Social Work, University of Kentucky, Lexington, KY, United States; ^3^Humane Law Enforcement, American Society for the Prevention of Cruelty to Animals (ASPCA^®^), New York, NY, United States; ^4^Strategy and Research, American Society for the Prevention of Cruelty to Animals (ASPCA^®^), New York, NY, United States; ^5^College of Social Work, University of Tennessee, Knoxville, TN, United States

**Keywords:** pet quality of life, human-animal bond, low-income veterinary clients, community veterinary medicine, Spanish-speaking adults

## Abstract

Perception of quality of life for cats and dogs of low-income Spanish and English-speaking veterinary clients attending problem focused or routine veterinary visits is an important area of focus for community based veterinary service providers. Using a qualitative approach, 50 New York City based American Society for the Prevention of Cruelty to Animals (ASPCA) veterinary clients completed semi-structured interviews as well as a survey about their perception of life with their pets. Veterinary clients shared both human-animal bond (HAB) related and quality of life (QoL) related factors in their daily experience of life with their pets. Results indicated that this demographic perceives QoL similarly to previous QoL research that either does not report sample demographics or reports sample demographics with more affluence. Moreover, 60% of qualitative excerpts included both HAB and QoL themes and 40% were discretely HAB or QoL. An analog single item 10-point scale measuring veterinary client perception of their pets QoL did not differentiate between sample demographics at a statistically significant level. Finally, pet QoL literature has not traditionally reflected diverse demographic identities of veterinary clients or widely included reliable and valid measures of the human-animal bond (HAB). These results support the importance of measuring the HAB when researching pet QoL and provide evidence that lower-income Spanish and English-speaking veterinary clients are similarly bonded and attentive to their pets as other demographics.

## 1 Introduction

Quality of Life (QoL) is a concept that has been discussed in healthcare since the mid 1900's (1–3). Decision making related to QoL has been important in human and veterinary health care settings alike (4). Many definitions of animal QoL have been proposed (5–8) however, there is yet to be a single, widely accepted definition (9, 10). Additionally, research has found that the human-animal bond (HAB) influences clients' perception of their pets QoL (11), however, measuring HAB with reliable and valid measures has been lacking in QoL literature (10). How clients perceive and give meaning to their pets behavior is subjective in nature (12) and likely influenced by their bond with the pet. This subjectivity can make it difficult to establish reliable and valid measures of pet QoL for the purposes of medical care decision making (10). Finally, little attention has been paid to how low-income clients may experience or describe their pets' quality of life (13, 14).

### 1.1 Quality of life and understanding medical outcomes

Some argue that animal QoL is synonymous with animal “welfare” (4, 8, 15–17) and much like the QoL definition, there is no single way that animal welfare is defined (18, 19). Traditionally, animal welfare has focused on preventing abuse and neglect, whereas animal QoL has focused on the thriving wellbeing of animals, including psychosocial (8, 20, 21) and emotional wellbeing (20, 21). The Five Freedoms and Five Domains highlight that in order to ensure that animals live happy, healthy lives, the negative aspects of their lives must be reduced and positive mental experiences must also be increased (22, 23). The Five Freedoms outline necessary and sufficient conditions for good animal welfare and quality of life. These include the freedoms from fear and distress, discomfort, pain, injury, and disease, hunger and thirst, and the ability to behave normally (24). In most of human healthcare QoL is assessed by self report. In veterinary medicine, much like pediatric medicine, a client is the proxy reporter of pet QoL. Ensuring positive mental experiences and reducing animal physical distress can all be found in a strong human-animal bond (HAB) that exists in responsible pet ownership (25). Veterinary client's emotions and perspectives about their pets QoL in times of illness has important implications for veterinarians who must rely on clients' assessments of pet QoL for determining if the animal's welfare is acceptable, what medical care is needed and if it is working, and also if an animal is coming to the end of its life (26).

### 1.2 The human-animal bond, quality of life, and animal welfare

Many QoL scales have been proposed. However, they are frequently limited by assessing a single dimension, such as a specific disease or species, which is not representative of general veterinary practice (9, 27). Studies that have specifically researched QoL dynamics, such as emotional wellbeing (6, 20, 21) operationalize QoL domains as vitality, comfort, and emotional wellbeing. Freeman et al. (28) describe QoL as physical, mental, emotional, and social functioning. Tatlock et al. (29) includes physical and non-physical factors in their description of QoL. In a recent systematic scoping review of nine measurement tools assessing QoL, Fulmer et al. (10) reported that common factors across assessments included activity level, desire of interaction, and appetite of the pet. What was not common across measurement tools was inclusion of complete client demographics or an assessment of the emotional attachment to the pet and how it affected perception of QoL.

### 1.3 Low-income clients and access to veterinary care

There are ~19 million pets living with families whose income level is below the United States (US) poverty line (30). Although research attention on the topic is growing (14), traditionally research exploring pet companionship for low-income families has been limited. Rauktis et al. (31) suggest that low-income clients enjoy the same pet-related advantages as income secure clients, including companionship, unconditional acceptance, and decreased social isolation. Despite potentially high levels of happiness and emotional investment, obtaining veterinary medical care for a pet may be limited when financial resources are scarce (14). LaVallee et al. (13) identified five common barriers to the accessibility of veterinary care: (1) the cost of veterinary care, (2) accessibility of care, (3) impaired veterinarian-client communication, (4) culture/language barriers, and (5) lack of client education. LaVallee et al. (13) also suggested that more research is needed on the effectiveness and efficiency of community medicine initiatives.

The efficacy of community medicine initiatives that serve low-income families is influenced by veterinary clients' perceptions of a pet's QoL and the HAB, both of which affect clients' medical decision-making (9, 13). For example, Rauktis et al. (14) presented the case of a single mother who stated that while she knew her daughter would never forgive her if they had to give up the pet or put it down because they couldn't afford an operation, they could not afford to put their finances at greater risk if the family pet needed back surgery. Another important term for the ways that family circumstances such as these affect the human-animal relationship is called Family Quality of Life (FQoL) (32). An earlier study of food insecurity in low-income households with animal companions found that food bank pantry volunteers believed that clients who did not have pet food were giving their meat and fish to their pets because owners viewed their companion animal as a family member and were committed to keeping them healthy (31). Arrington and Markarian (30) point out that, like human food deserts, there are animal resource deserts where entire neighborhoods lack veterinarians or pet supply stores.

The experience of poverty has been shown to have associations with human neurodevelopment (33) and can be associated with an attentional bias toward threat (34). This attentional bias, the over-tendency to observe external stimuli as threatening, could have an impact on how clients perceive their pet's health and behavior. Serpell (12) also argues that the subjectivity of the HAB makes it difficult to determine animal welfare from an owner's perspective. The experience of poverty and how this impacts brain development (33), and perception of self-efficacy (35) are important considerations in understanding how the nature of the HAB for low-income veterinary clients may impact clients' perspectives of pet QoL.

The present study explored the perception of pet QoL for Spanish and English-speaking clients with dogs or cats who were seeking routine or problem-focused veterinary treatment for their pets. The first aim was to explore how low-income veterinary clients perceive their pet's QoL. The second aim was to explore differences in subjective QoL scores based on species (cat or dog), whether the pet had a medical problem or was receiving routine care, and the preferred language of clients (Spanish or English).

## 2 Materials and methods

In this qualitative study, data were collected through semi-structured interviews from English and Spanish-speaking veterinary clients with dogs or cats who utilized services at two American Society for the Prevention of Cruelty to Animals (ASPCA) locations (a community clinic and a veterinary hospital) in New York City (NYC) between July 20, 2021 and September 28, 2022. To access ASPCA services, clients must live in New York City and have a self-reported annual income under $50,000. The ASPCA community clinic offers subsidized preventatives (e.g. vaccines and parasite control) and basic medical care (e.g. skin condition care, respiratory illness treatment, medical grooming, etc). The hospital provides subsidized urgent or emergency services for qualifying medical conditions with good prognosis, such as orthopedic surgery. Patients with poor prognoses and comorbidities, such as cancer or diabetes, are not eligible for care but may be treated palliatively at a low cost to the owner. In appropriate circumstances, no-cost euthanasia may be offered.

### 2.1 Participant recruitment

After setting an ASPCA veterinary appointment, clients were contacted by phone and text message inviting them to participate in the research study. If they agreed to be in the study, clients participated in a semi-structured interview via Zoom ~1–5 days before the pet veterinary appointment at the community clinic or between treatment and recheck appointments at the ASPCA hospital.

### 2.2 Data collection

Six trained research assistants (four English-speaking and two bi-lingual Spanish/English-speaking) conducted individual semi-structured interviews with clients. Interview data were collected via Zoom web-based meetings. The use of online technology, such as telephone and Zoom-based interviews, have been noted as different but equal methods for data collection (36–40) in qualitative research. Semi-structured interview questions focused on clients' experiences living with and caring for their pets generally (“*What is a day like for you and your pet?*”), as well as their perception of their pets' QoL (“*How would you describe your pet's quality of life?”*). The interview included a 1–10 point Likert scale figure with 1 indicating “Terrible” QoL and 10 indicating “Excellent” QoL. Clients were asked to describe what they would observe if their pet's QoL was a 1, 5, and 10. At the conclusion of the interview, clients were provided a survey link to complete demographic questions, reasons for the upcoming veterinary visit, and a numerical ranking of their own pets' current QoL.

### 2.3 Data analysis

Veterinary client interviews were analyzed using an interpretative phenomenological analysis (IPA) framework. There are two parts to IPA: the phenomenological component and the interpretative component (41). The phenomenological component explores how a research participant understands a phenomenon- in this case, a pet's QoL, and the interpretative component contextualizes what these understandings mean to the client and to the phenomenon as a whole across the sample. A coding team of four animal-related professionals (one of whom was fluent in Spanish) and six social work professionals (one of whom was fluent in Spanish and one of whom had over 20 years of experience in the veterinary field) bracketed their own experience with the phenomena being explored prior to data analysis (42). The team then reviewed a set of the same transcripts, coded notable transcript excerpts, and used memos to track ideas, perspectives, and questions about the codes. The team then met to discuss memos and agreed on emergent codes observed in the data. Thereafter, each client interview was coded by two researchers, with a 3rd researcher coding interviews where the first two coders diverged substantially in their codes. The coding team met frequently throughout the analysis to discuss applied codes and memos until consensus was reached. Using Dedoose Version 9.0.17, (2021, Los Angeles, CA: SocioCultural Research Consultants, LLC; www.dedoose.com), the research team applied codes to the interviews. After coding, an interpretative and thematic analysis of the codes was used to identify sub-themes and overarching themes observed in the data [see Spiers and Riley (43) for how IPA and thematic analysis can be complementary methods].

Descriptive statistics were used to report on sample demographics and post-interview QoL scores for dogs and cats, Spanish and English-speaking clients, and problem-focused vs. routine pet visits. SPSS v. 26 was used for quantitative data analysis (IBM Corp. Released 2019. IBM SPSS Statistics for Mac, Version 26.0. Armonk, NY: IBM Corp). This research was approved by the University of Tennessee Human Subjects Institutional Review Board UTK IRB-20-05843-XP.

## 3 Results

### 3.1 Sample

**Figure 1:**
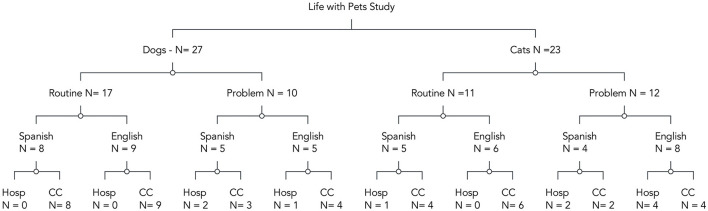
Diagram of total samples per subgroup *N*'s.

In all, 50 cases were analyzed. Fifty-six percent (*N* = 28) of clients reported their primary language as English and 44% (*N* = 22) as Spanish. Nearly three-quarters (74%, *N* = 35) self-identified as Hispanic (*N* = 35). Fifty-four percent (*N* = 27) of the pets were dogs and 46% (*N* = 23) were cats. Fifty-six percent (*N* = 28) of appointments were routine visits, and 44% (*N* = 22) were problem visits (see Figure 1). Eighty percent (*N* = 40) of visits were in the community clinic, and 20% (*N* = 10) were at the ASPCA hospital. More than half of clients (52%, *N* = 26) reported an annual income below $15,000 (see Table 1).

**Table 1:** Demographics of study population.

**Variable**	***N* (= 50)**	**Percent**
**Age of veterinary client**		
21–28	8	16%
29–36	15	30%
37–44	6	12%
45–52	8	16%
53–604	9	18%
61+	4	8%
**Gender of veterinary client**		
Female	42	84%
Male	6	12%
Non-binary	1	2%
Missing	1	2%
**Race/ethnicity of veterinary client 1**		
Reported Hispanic Ethnicity only	28	56%
Black	9	18%
White	6	12%
Reported country of origin	5	10%
No response	2	4%
**Primary language of veterinary client**		
English	28	56%
Spanish	22	44%
**Annual income of veterinary client**		
Less than $15,000	26	52%
$15,000–$34,999	12	24%
$35,0000–$49,999	8	16%
$50,000–$74,999	1	2%
$75,000–$99,999	1	2%
Not sure	2	4%
**Number of people in household including the veterinary client**		
1	9	18%
2	17	34%
3	10	20%
4	3	6%
5	7	14%
6+	4	8%
**Pet type**		
Cat	23	46%
Dog	27	54%
**Location of visit**		
Clinic	40	80%
Hospital	10	20%
**Type of visit**		
Problem visit	22	52%
Routine visit	28	48%

### 3.2 HAB and QOL themes

Qualitative analysis resulted in 2,720 excerpts with 32 codes across 50 semi-structured interviews. Excerpts could have more than one code applied. Results of the qualitative analysis of these excerpts produced two main themes and four sub-themes. Two sub-themes were focused on the QoL: animal mental/physical condition (*N* = 5 sub codes) and daily rhythms and habits (*N* = 8 sub codes). Two sub-themes were focused on HAB: nature of attachment (*N* = 8 sub codes), and human/pet family pet characteristics (*N* = 5 sub codes). The qualitative analysis also produced six codes that related to a 10-point scale shown to participants about perspectives on QoL.

#### 3.2.1 QoL theme

- Animal mental and physical condition sub-theme; there were 1,184 excerpts regarding the animal's mental and physical condition. Clients commented on their pets' behavior (*N* = 552) “*Then, he follows me actually. That's what he does, follow me everywhere I go,”* health concerns (*N* = 495) “*...he didn't want to eat, he didn't want to drink water, he was going to the bathroom very often. Those were the first symptoms we saw when he was sick,”* pet preferences (*N* = 221), “*She have her hiding spots. She just chills out in there, until she's ready to come out for her food,”* hygiene (*N* = 132), “*I brush him, I bathed him, I brushed him. I have him nice and ready for tomorrow. He likes to be bathed*,” pets' breed (*N* = 35), “*She's a German Shepherd and terrier.”*
- Daily rhythms and habits sub-theme; There were 1,099 interview excerpts that focused on pets' daily rhythms and habits. Clients commented about their pets' nutrition (*n* = 449), “*She doesn't want to eat sometimes, there's something about the food,”* play (*n* = 321), “*She likes to play with her little toys. She grabs them and she just tossed, throws them around,”* sleep (*n* = 227), “*Right now, she is asleep. She sleeps a lot. She sleeps during the day but she doesn't have specific sleep times. She's quiet at home and she falls asleep,”* exercise (*n* = 150), “*He used to run around a little bit more than he used—than he does now, just randomly get the cat zoomies*,” elimination behaviors (*n* = 145), “*She used to only pee when we took her outside, but later she started going here in the apartment a lot,”* undesirable behavior (*n* = 135), “*He had a scratching post and he was not using it. He was scratching everything else that was wood except that,”* vocalization (*n* = 121), “*He has separation anxiety, so sometimes when I leave he barks a lot,”* training (*n* = 70), “*She'll see wires…She'll bother them, but I'll tell her somethin' without even touching her, and she knows that that's the wrong thing to even be doin', so she'll just walk away.”*

#### 3.2.2 HAB theme

- Nature of attachment sub-theme; There were 1,173 excerpts that focused on the nature of attachment between pet owner and pet. Clients commented about the naming and familial roles of their pets (*n* = 537), “*I'm very grateful for having my son [Tommy]. I love it very much. He is my life; he is my life,”* empathic interpretation/action of pet emotion (*n* = 470), “*Well, I think that he feels happy, because when we get home he wags his tail, he likes to be with us. If we leave, he looks for us, and everyone wants to be with him,”* physical touch (*n* = 206), “*And I like her above all because she's very affectionate; she lets everyone pet her. Especially since I have a little child in here. It's good to have a pet like that,”* pet owner protectiveness (*n* = 203), “*She had a hard life and I make it my goal for her to never have a hard life ever again,”* benefits of being a pet owner (*n* = 130), “*You forget about stuff—you're running around, for whatever reason your stress level is high, but she has helped me relieve my stress because she keeps me busy and playing with her toys and I feel like a kid again. I think it would be good if people could keep pets to feel much better,”* sacrifices made for the pet (*n* = 86), “*I've been selling my jewelry, things I had at home, in order to cover… the food and grooming for the dogs,”* emotional support from the pet (*n* = 68), “*He's actually like my—my little dogs are—my little dogs are certified, and they're my emotional support dogs,”* and finance (*n* = 51) “*we split the cost of buying the cat and everything. We end up splitting the financial burden of certain things. Especially doing these emergency vet visits and things, I've definitely had assistance from him on that front.”*
- Human-pet family characteristics; There were 874 excerpts that focused on human/pet and family characteristics. Clients remarked about seeking professional help for their pet (*n* = 300), “*She doesn't really have pain or anything. It's just I just noticed that the spots are there, so I don't know 100 percent if something's causing them or if they're like a birthmark of some sort, so I just wanted to take her again just to be 100 percent sure,”* having multiple pets (*n* = 222), “*I actually have two little dogs,”* having multiple people living in the home (*n* = 219), “*I have three children and he brings a lot of joy to our family,”* the pet being rescued (*n* = 134), “*Yes, I got Tommy because it happens that in my grandmother's building there was a man whose cat gave birth all the time. Then, one day, I grabbed Tommy. And this is Tommy, in the basement where my grandmother lives*,” and, history of their own pet keeping (*n* = 122) “*I've had dogs, fish, hamsters, [laughter] that type of good stuff.”*

#### 3.2.3 Overlap and discrete HAB and QoL excerpts

**Figure 2:**
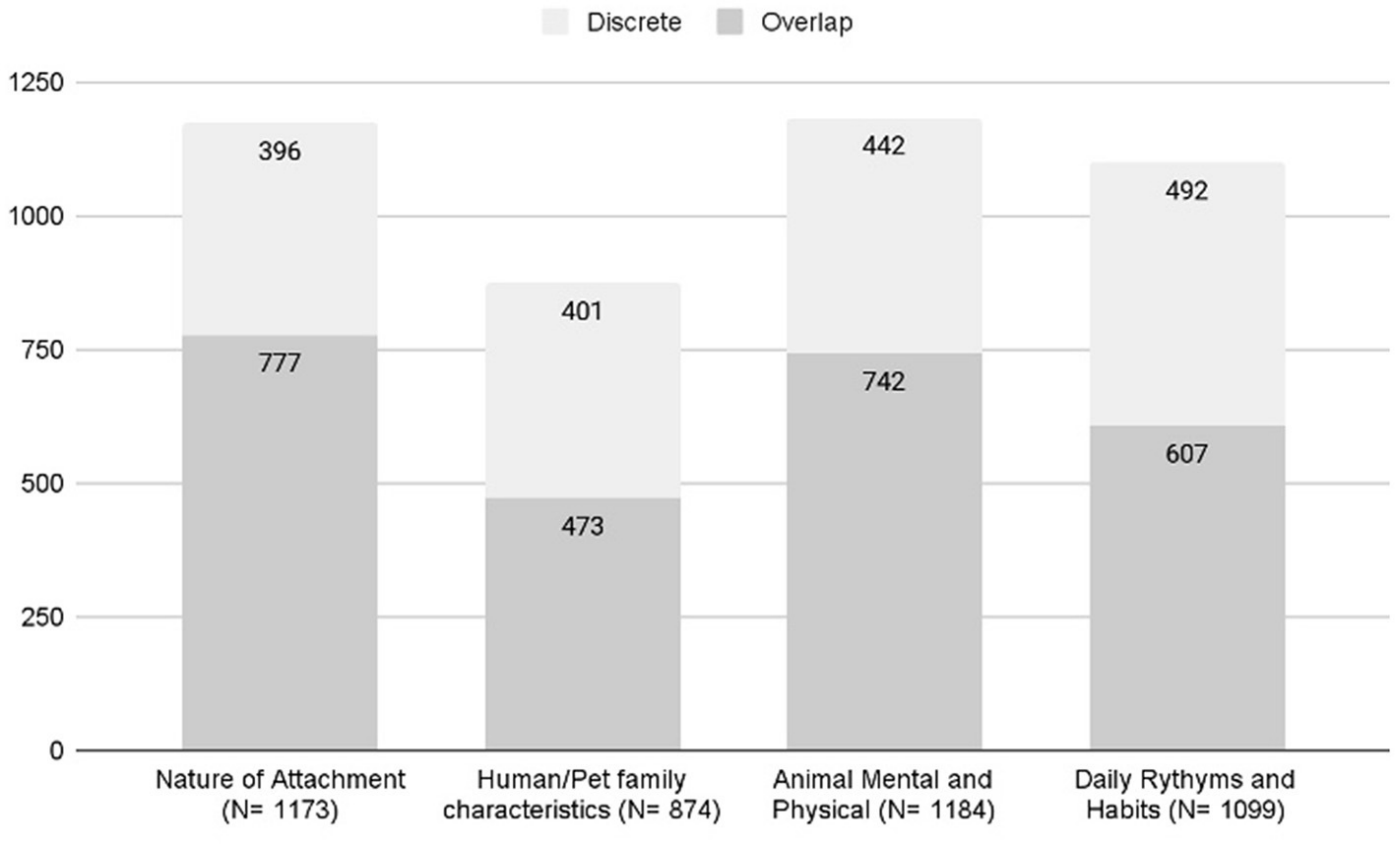
Overlap and discrete QoL and HAB sub-themes.

There were 1,731 excerpts coded with QoL themes and 1,697 coded with HAB themes. Sixty percent of excerpts (*n* = 1,033) were coded with both QoL and HAB themes. The codes with the strongest co-occurrence were animal behavior × empathic interpretation/action of pet emotion (*n* = 187), health concerns × empathic interpretation/action of pet emotion (*n* = 146), health concerns × professional help seeking (*n* = 154), and naming and familial interaction × nutrition (*n* = 116). Forty percent (*n* = 698 of 1,731) of QoL excerpts were discrete with no HAB codes and 39% (*N* = 664 of 1,697) of HAB excerpts were discrete with no QoL themes (see Figure 2 for discrete sub-theme excerpt counts). The child themes where more than 50% of excerpts were discretely coded as QoL were: breed 66% (*n* = 23 of 35), undesirable behavior 56% (*n* = 76 of 135), training 54% (*n* = 38 of 70), and sleep 53% (*n* = 121 of 227). The child themes where more than 50% of excerpts discretely coded as HAB were: history of pet keeping 86% (*n* = 105 of 122), rescue pet 71% (*n* = 95 of 134), emotional support 57% (*n* = 39 of 68), benefits of pet ownership 53% (*n* = 69 of 130), and finance 53% (*n* = 27 of 51; see Figure 2).

### 3.3 Perception of QoL scores at levels 1, 5, and 10

**Figure 3:**
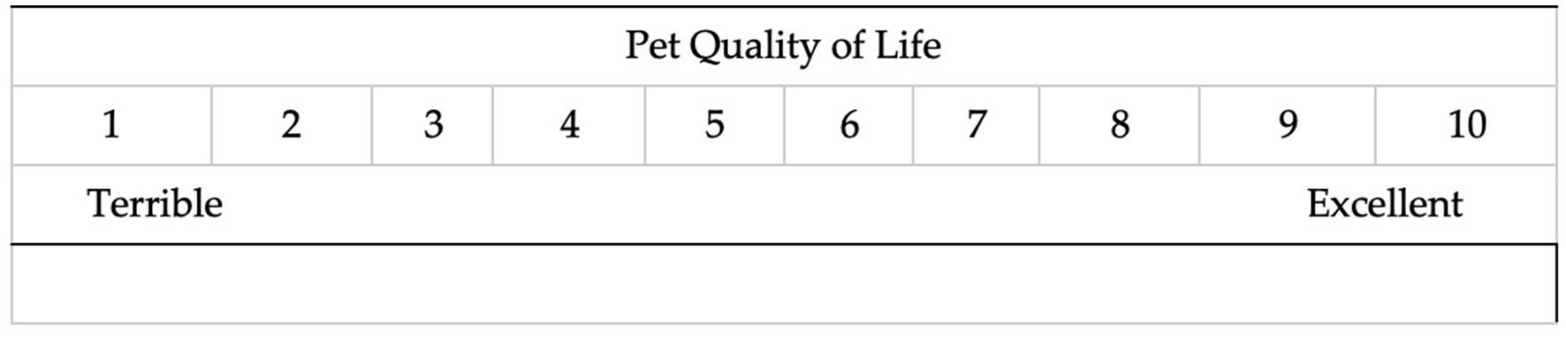
QoL scoring tool.

Clients were shown a 1–10 scale ranging from 1 = “Terrible” and 10 = “Excellent” (see Figure 3). They were asked to describe how their pet's QoL would be at a level 1, level 5, and level 10. Discrete QoL excerpts across 1, 5, and 10 included elimination behavior, exercise, nutrition, play, sleep, health concerns, and hygiene. Discrete HAB excerpts across 1, 5, and 10 included empathic perception/action toward pet emotion (see Table 2).

**Table 2:** Veterinary client perception of pet QoL at 1, 5, 10: codes and example excerpts^↑^.

**Sub codes**	**QoL 1 (*N* of example excerpt)**	**QoL 5 (*N* of example excerpt)**	**QoL 10 (*N* of example excerpt)**
QoL elimination behavior	*N* = 5 excerpts	*N* = 3	*N* = 1
	*I think that if he was in a really bad place, I think I would probably notice him peeing everywhere too*	*They don't have a dog life. They just stay in the apartment all day, or in the house all day. All they have, they put 'em in the backyard, and they don't take 'em anywhere. Go do your need in the backyard, and that's it*	*She wouldn't have diarrhea and stuff like that, so no health issues I would say at a 10*
QoL exercise	*N* = 17	*N* = 5	*N* = 5
	*Being neglected, like not bein' taken care of the way he's supposed to be. That's a one. Lettin' him go hungry. Not walking him. Not socializin' him. That's a one*	*I would see him sad, dispirited, maybe? Restless. Let's say, maybe he wouldn't eat. Uh-huh. He wouldn't be very active*	*I really think—and he would probably have more space. He'd probably be running around a little more*
QoL nutrition	*N* = 19	*N* = 18	*N = 9*
	*Well, starving and such*	*Not a lot of energy, but yet eating. Not being that active. Eating, but not as much*	*It would be an excellent level, he would have all the amenities he needed, he would eat well, he would behave well with people*
QoL play	*N* = 5	*N* = 9	*N* = 11
	*Just very triggered I would say if she had a one quality of life. Not wanting to play*	*He probably would not be very interested in playing with the things that he normally likes to play with*	*A 10 would be—yeah, excellent. Runnin' around, jumpin' on the couch, jumpin' on the other animals, just doin' stuff*
QoL sleep	*N* = 3	*N* = 3	*N* = 3
	*A one will say his energy will be draining. He would be sleeping most of the time. He'll be sad, I mean, one sounds horrible, so, yeah*	*That's what it is, when she's not feeling the way she should, she becomes—she goes in her solitary, she has a bed, she'll go in her bed*	*Yes, she's playful, she plays with her other siblings. She'll sleep with the other ones*
QoL health concerns	*N* = 23	*N* = 19	*N* = 9
	When she first came to me, she was full of fleas, underweight, shivering, homeless, terrified. Also that one Friday night when her wound had gotten so bad, she couldn't walk. She was limping. She still had an appetite, which was crazy. Friday, that one Friday night was absolutely terrible	*I think probably in terms of her weight, she wouldn't probably be as solid as she is and active, and she wouldn't have her appetite. I think she would probably be—she would have an appetite but not much, and she wouldn't be very active at all. She would be doing a lot of sneezing and having breathing issues so much, that you hear, like when a person has bronchitis, that's how she was going for a minute*	*Well, she wouldn't have any issues with her lungs or upper respiratory. She wouldn't have that. She wouldn't have that cyst on her head. She wouldn't have issues in terms of her sneezing or wheezing or anything like that. She'd probably be in more mischief than normal, than she normally is [laughter], a whole lot more, be a whole lot worse than what she gets into. She'd really be tearing up the house and stuff like that. Her condition would be great. Her appetite would be there. She wouldn't have diarrhea and stuff like that, so no health issues I would say at a 10*
QoL hygiene	*N* = 5	*N* = 3	*N* = 2
	*I think she would be dirty, poorly fed, I don't know, sick*	*they're problematic with bathing*	*I can't always take him to get him trimmed, because my dog is one of those that needs grooming. We used to pay but now we bought the machine and we do it. I only take him to get his nails cut. That's why I say that for me, a 10 is when someone provides the dog with a lot of care*
HAB empathic perception/action based on pet emotion	*N* = 8	*N* = 5	*N* = 4
	*Honestly, that would be quite bad having a dog when people even abuse them, they hit them. There are people who have dogs only to breed them and for me that is quite awful*	*Mm, they wouldn't give him affection, they wouldn't take him to the doctor. That would be a bad life, because you have to take him to the doctor. There are people who give their pets love, they give their pet affection, but they don't take them to the doctor, and they need to go to the doctor*	*A dog that's well fed. A dog that is well taken care of. A dog that is taken to the vet for his shots, and a dog that is watched over and taken out to activities. My dogs, they went to the Pride Parade. They had fun. A dog that has fun. A dog that is allowed to be a dog and not expectin' them to be like little babies, little kids, and behave like little kids. They are dogs at the end of the day, and they have to be—and they count on the person. They count on me, and I have to be there for them because they didn't ask me to bring 'em in. Now they count on me, and I have to be there. That's a 10 for a dog. A dog that all his needs are met*

^↑^Clients were shown Figure 2 with 1 = Terrible QoL and 10 = excellent QoL and asked to describe their pet at a 1, a 5, and a 10.

### 3.4 Post-interview veterinary client pet QoL scores

There were no statistically significant differences in the QoL scores veterinary clients assigned to their pets in the post interview survey (see Table 3).

**Table 3:** Post interview veterinary client QoL scores.

**Variable (*N*)**	**Median QoL**	**Range QoL**
Total sample (*N* = 50)	9.5	5–10
**Animal**		
Dog (*N* = 27)	10	5–10
Cat (*N* = 23)	9	7–10
**Visit type**		
Routine (*N* = 28)	10	7–10
Problem (*N* = 22)	9	5–10
**Location**		
Community clinic (*N* = 40)	10	5–10
Hospital (*N* = 10)	9	7–10
**Primary language**		
English (*N* = 31)	9	6–10
Spanish (*N* = 19)	10	5–10
**Ethnicity**		
Hispanic (*N* = 35)	10	5–10
Non-Hispanic (*N* = 13)	9	7–10
Missing (*N* = 2)	9	8–9
**Household income**		
Less than $15,000 (*N* = 26)	9	6–10
$15,000-$34,999 (*N* = 12)	9.5	5–10
$35,000 to $49,999 (*N* = 8)	10	7–10
$50,000 to $99,999 (*N* = 2)	10	10
Unsure (*N* = 2)	9	8–10
**Household income dichotomized**		
Under $15,000 (*N* = 26)	9	6–10
$15,000- $49,000 (*N* = 20)	10	5–10
**Language-visit-animal type**		
English problem dog (*N* = 5)	9	7–10
English problem cat (*N* = 8)	8.5	7–10
English routine cat (*N* = 6)	9.5	8–10
English routine dog (*N* = 9)	10	6–10
Spanish problem cat (*N* = 4)	9.5	9–10
Spanish problem dog (*N* = 5)	9	8–10
Spanish routine cat (*N* = 5)	10	7–10
Spanish routine dog (*N* = 8)	10	5–10

## 4 Discussion

The aims of this research were to explore how low-income veterinary clients perceive their pet's QoL and explore differences in subjective QoL scores based on species (cat or dog), whether the pet had a medical problem or was receiving routine care, and the preferred language of clients (Spanish or English). Our results indicate that when asked to speak about life with their pets, lower-income Spanish and English-speaking veterinary clients' perspectives span across two QoL themes (animal physical and mental condition, daily habits and rhythms) and two HAB themes (nature of attachment, and human/pet family characteristics). This sample of low-income, predominantly Hispanic, veterinary clients discussed the same QoL factors found in other QoL research. There were not substantial differences in what veterinary clients discussed between client preferred language, species, or type of visit within our sample either. Lastly, there was a significant overlap between qualitative excerpts that included both QoL and HAB themes.

### 4.1 Veterinary client demographics

Although there is recognition that pet QoL is influenced by veterinary clients' perspectives as the “proxy” or “surrogate” for their pet (44), assessing client demographics in the development of instruments to measure QoL is missing (4, 6, 28, 29, 45–47) or highly limited (48, 49). Additional research examining pet QoL included samples of mostly women, employed, and in secure housing (50–53). Seventy-six percent of the clients in this sample self-identified as low-income households. Our findings support that low-income Spanish and English-speaking clients experienced high levels of emotional investment in their cats and dogs. LaValle et al. (13). reports that when obtaining veterinary services is a financial strain it can negatively affect QoL of both humans and animals. Our findings also support this. A veterinary client described this intersectionality of the financial concern of owning their pet and their HAB by stating, “*I thought it was too expensive, so no. We didn't have the resources to get a pet, but they gave it to her and I thought, I'm going to go ask it's expensive, if it's a big expense, but we ended up getting attached to him.”* Another client shared her commitment to caring for her pet by saying, “*Currently I know he is happy, he is well fed, he is clean, we always keep him clean. One way or the other—as I mentioned, I've been selling my jewelry, things I had at home, in order to cover my daughter's gas costs and the food and grooming for the dogs because the truth is that it's been quite difficult these past two years.”* Our findings confirm that lower income clients experience the HAB and QoL concerns just like clients with higher means that have comprised most demographic samples in QoL research. Determining if there are meaningful differences in how low-income and higher-income clients experience the HAB and QoL would require a sample with a larger distribution of incomes represented. This would be helpful in informing low-cost veterinary providers about the unique needs of their clients and how those needs may impact pet QoL. For instance, all clients may make sacrifices for their pets, however lower-income clients' sacrifices may be more related to basic human needs like gas or food, while higher-income clients' sacrifices may be related more to time spent giving up pleasure like a vacation in order to stay with a sick pet.

### 4.2 HAB and measuring QoL

A strong HAB has been associated with higher levels of physical health, emotional wellbeing, and positive social interactions for pets (11). There is also recognition that the HAB may increase during times of pet illness (54, 55). On the human side, however, there is evidence that strong bonds with pets can also create caregiver burden and mental health challenges for clients when pets become ill (26, 56). This human stress can have an impact on how veterinary clients perceive their pet's QoL. Although effort has been put toward creating instruments that purely measure health-related quality of life in pets (6, 7, 28, 57), the impact that the HAB may have on clients' perception of pet health could be impacted by personal experience of poverty (33, 35), client personality differences (58), and client quality of life factors also known as Family Quality of Life (49, 59, 60). This is very important for veterinary professionals to consider when treating animals of lower in-come veterinary clients. Although these results suggest HAB and QoL for this population is similar to other more affluent groups there may be differences in how this is expressed in the veterinary client patient relationship. For instance, Family Quality of Life factors such as the stress of poverty and level of ability to pay for veterinary care, as well as pet caregiver burden, likely impact the HAB and perception and reporting of pet QoL to the veterinary team.

Traditionally, this HAB-QoL relationship in pet QoL research has been missing. For instance, Noble et al. (6), Tatlock et al. (29), and Freeman et al. (28) did not incorporate an assessment of HAB in methodology or integrate it into discussion of findings. Lavan (48) acknowledges psychosocial QoL factors can be impacted by their human research subjects' attentiveness to their pets in the discussion section, however, the scale itself did not attempt to measure the HAB. Bijsmans et al. (47) recognized in their discussion that the unique nature of the client-cat bond is crucial in interpreting the results of their Cat QoL questionnaire; however, the tool did not integrate this sentiment into the items.

Our results indicate that 60% of the qualitative excerpts of clients' verbalizations about lives with their pets shared an overlap between both the HAB and QoL topics. This supports what both Bijsmans et al. (47) and Lavan (48) mentioned as a relevant consideration in QoL research. Fortunately, recent investigation into QoL has started to incorporate reliable and valid measures of HAB. For instance, Piotti et al. (5) assessed clients' pets QoL in Italy (IT) during the COVID-19 pandemic using both the Milan Pet Quality of Life Instrument (MPQL) and the Lexington Attachment to Pets Scale (LAPS). Their findings suggest a positive correlation between “general attachment” and a heightened desire for client-pet interactions. This increased level of engagement may indirectly positively impact the overall wellbeing of pets (5). Testoni et al. (53) evaluated Italian clients' QoL by employing the HHHHMM QoL scale and LAPS and also gathered qualitative data about clients' grief. Findings indicated that clients living alone are more inclined to anthropomorphize companion animals, particularly dogs, attributing traits associated with social connectedness. These are examples of important progress in examining QoL by integrating client demographics and reliable and valid HAB instruments into the methodology.

In our findings, QoL-HAB sub-codes that greatly overlapped included pet owner empathic perception/actions related to pets' emotions (HAB) and its co-occurrence with both animal behavior (QoL) and nutrition (QoL). These findings suggest that how clients interpret their pet's emotions is important to consider in how they describe their pet's behavior and nutritional habits. Sub-codes from our research that may differentiate between HAB and QoL include the QoL sub-codes of animal breed, type of undesirable pet behavior, training, and sleep. Sub-codes such as sleep habits and the pet's undesirable behaviors (breaking things, scratching household items, excessively licking client, etc), may be topics that are more directly tied to QoL and less influenced by HAB. Topics such as the history of pet keeping, rescue pets, emotional support from the pet, benefits of pet ownership, and finances were discreetly associated with HAB. Interestingly, in a study assessing both human and dog characteristics, history of pet keeping was a strong predictor of the level of attachment within the dog and client dyads (50), supporting our findings that history of pet keeping is discretely related to HAB. Considering these discrete QoL or HAB topics in future QoL measurement efforts may be helpful in assessing health-related quality of life without the confounding influence of the HAB.

Another way client perception of QoL was assessed was through a post-interview survey that asked veterinary clients to provide a score for their pet's current QoL using the 1 item scale used in the interview (1 = Terrible to 10 = Excellent). Veterinary client post-interview quantitative scores did not show meaningful differences between problem and routine visits, cats or dogs, primary language of the client, or income levels. Although the 1-item scale in this study did not identify statistically significant differences in core study demographics, this is likely due to the smaller sample size typical of qualitative research. The use of 1-item assessments has been found to be an effective measurement approach (61). Single-item measures have many benefits, such as being more efficient and satisfying for users to take and so further effort to develop this method is warranted (61). Future research could explore anchoring this 10-point scale with qualitative words that clients reported they would observe in their pet if their pet was at a QoL 1 = “starving” “peeing everywhere”; QoL 5 = “solitary” “lack of appetite” or QoL 10 = “playful” “sleeping with others” (see Table 2).

For the wellbeing of both humans and animals it is important to comprehend the nature of the relationship between mental health and the human-companion-animal relationship (60, 62, 63), as well as the subjective experiences of pet QoL (26). These “intrapersonal” factors could impact client reports of treatment outcomes and efficacy with the veterinary team. For instance, Rodger et al. (57), found a 40% disagreement between client and veterinary clinician assessment of pet health-related QoL, and acknowledged pet owner factors such as “mood” as important in assessing QoL. Future research should include client demographics as well as reliable and valid measures of HAB to continue discovering how these factors may impact client perception of pet QoL. Moreover, studies that include a wide range of demographics would be essential in exploring if differences exist in how HAB and QoL are exhibited in the veterinary-client-patient relationship. This knowledge will afford veterinary professionals the knowledge needed to uphold their public health duty caring for pets in households from this population level demographic.

### 4.3 Study limitations

The limitations of this research include restriction of range of the 10-point QoL scale which may have impacted the ability of the scale to discern QoL differences between pet owner demographics. Additionally, the question about race and ethnicity was open-ended, resulting in variability in how these data were reported and therefore results may not provide a complete assessment of clients' racial and ethnic backgrounds. While the sample size for the quantitative analyses was modest and no statistically significant differences were found, the actual differences in medians between the groups was only a single point on the QoL scale. This difference is not clinically meaningful and therefore statistical power is not an important consideration. It is possible that some veterinary clients were not able to participate due to the digital divide causing lack of access to technology for low-income communities (64).

## 5 Conclusions

Low-income Spanish and English-speaking veterinary clients of both well and ill cats and dogs discussed their strong HAB and concern for their pets QoL. A single item 10-point QoL scale may lack the ability to identify perceived QoL differences between pets having problems and pets having routine care, however future research could explore expanding the range and adding qualitatively descriptive anchors on a single-item scale. Pet QoL research suggests that the HAB influences QoL and our results support those findings with 60% of qualitative excerpts including both HAB and QoL themes. The bond clients have with their pets is connected to their own emotional state, especially when a pet is sick or when clients face barriers to accessing care. Finally, our findings suggest that lower-income Hispanic and non-Hispanic clients have similar QoL and HAB thoughts, experiences, and concerns as those studied in other QoL research with more affluent samples.

## Data availability statement

The datasets presented in this article are not readily available because all data pertaining to the manuscript cannot be shared in accordance with the confidentiality promise to study participants. Requests to access the datasets should be directed at: ES, estrand@utk.edu.

## Ethics statement

The studies involving humans were approved by the study was approved by the Institutional Review Board of The University of Tennessee Knoxville (IRB-20-05843-XP; approved 4/06/21). The studies were conducted in accordance with the local legislation and institutional requirements. The ethics committee/institutional review board waived the requirement of written informed consent for participation from the participants or the participants' legal guardians/next of kin because Data were collected in an anonymous manner so having names connected to informed consent would have made data identifiable.

## Author contributions

ES: Conceptualization, Formal analysis, Funding acquisition, Methodology, Project administration, Supervision, Writing – original draft, Writing – review & editing. KS: Conceptualization, Data curation, Formal analysis, Methodology, Project administration, Writing – original draft, Writing – review & editing. HW: Conceptualization, Data curation, Methodology, Writing – review & editing. AH: Conceptualization, Data curation, Writing – review & editing. VA: Conceptualization, Project administration, Resources, Writing – review & editing. LM: Conceptualization, Funding acquisition, Supervision, Writing – review & editing. PL: Writing – review & editing. CW: Formal analysis, Writing – original draft, Writing – review & editing. MK: Writing – original draft, Writing – review & editing. HE: Conceptualization, Data curation, Project administration, Writing – original draft. KM: Data curation, Formal analysis, Methodology, Project administration, Writing – review & editing. MS: Conceptualization, Resources, Writing – review & editing.
